# Frame-based stereotactic biopsies using an intraoperative MR-scanner are as safe and effective as conventional stereotactic procedures

**DOI:** 10.1371/journal.pone.0205772

**Published:** 2018-10-23

**Authors:** Jan-Oliver Neumann, Benito Campos, Bilal Younes, Martin Jakobs, Christine Jungk, Christopher Beynon, Andreas von Deimling, Andreas Unterberg, Karl Kiening

**Affiliations:** 1 Division Stereotactical and Functional Neurosurgery, Department of Neurosurgery, University Hospital Heidelberg, Heidelberg, Germany; 2 Department of Neurosurgery, University Hospital Heidelberg, Heidelberg, Germany; 3 Department of Neuropathology, University Hospital Heidelberg and, Clinical Cooperation Unit Neuropathology, German Cancer Research Center (DKFZ), and DKTK, Heidelberg, Germany; Johns Hopkins School of Medicine, UNITED STATES

## Abstract

**Background:**

Frame-based stereotactic biopsy (FBSB) is a minimally-invasive and effective procedure for the diagnosis of brain lesions and will likely gain clinical importance. Since FBSB procedures comprise a variety of imaging and sampling methods, it is necessary to compare the safety and effectiveness of individual techniques.

**Objective:**

To assess the safety and effectiveness of FBSB using 1.5T iMRI as a one-stop procedure under general anesthesia without intraoperative histological examination.

**Methods:**

In this single-center, retrospective analysis, 500 consecutive FBSBs using iMRI were compared to a historic control of 100 biopsies with traditional workflows (computed tomography (CT) with MRI image fusion). All procedures were performed under general anesthesia. Data on surgical procedures, pre- and postoperative neurologic patient status, complications and diagnostic yield were extracted from clinical records.

**Results:**

Complication rates and diagnostic yield showed no significant differences between both groups. Mortality was 0.6%, 95% CI = [0.12%, 1.74%], in the iMRI and 0.0% [0.00%, 3.62%], in the control group with a morbidity of 5.4% [3.6%, 7.8%] and 6.0% [2.2%, 12.6%] and a diagnostic yield of 96.8% [94.9%, 98.2%] and 96.0% [90.1%, 98.9%]. Mean procedure duration was 124 [121, 127] minutes using iMRI and 112 [106, 118] minutes in the control group.

**Conclusion:**

FBSB using 1.5T iMRI under general anesthesia is a safe and effective procedure and is equivalent to traditional stereotactic workflows with respect to complication rate and diagnostic yield.

## Introduction

Frame-based stereotactic biopsy (FBSB) is a minimally-invasive and effective procedure for the diagnosis of brain lesions. With the introduction of molecular diagnostics and targeted therapies, FBSBs will likely gain clinical importance. Since FBSB procedures can comprise a variety of imaging and sampling methods, it is necessary to compare the safety and effectiveness of individual techniques.

FBSBs can be performed under general or local anesthesia as well as combining stereotactic imaging under local and subsequent tissue sampling under general anesthesia. In addition, only parts of the overall procedure will take place in the OR and the transfer of the patient from the imaging site to the OR can make FBSB a time-consuming process. Furthermore, some departments routinely employ intraoperative histological tissue sampling, which adds to the local infrastructure requirements and the procedure duration.

Combining FBSB with intraoperative MR-imaging (iMRI) might offer several advantages, such as the possibility to perform the entire stereotactic procedure under general anesthesia in the OR, while sparing the patient potentially harmful radiation exposure. Spatial image distortion has been a concern in solely MRI-based stereotactic procedures and experimental studies have demonstrated deviations of trajectories of one voxel or even more [1–3]. Recently, we reported that 3D MRI image distortion correction may at least in part overcome image distortion artifacts [4].

As a consequence, we hypothesize that iMRI-based biopsies are as safe and effective as CT/MRI fusion-based procedures and present our experience with a series of five hundred, consecutive iMRI-based FBSBs. We will compare these procedures to a historic control series of one hundred FBSBs, which were performed with a conventional imaging workflow.

## Material & methods

### Study design

In this single-center, retrospective analysis we examined five hundred consecutive cases of FBSBs where stereotactic imaging was performed in an intraoperative MR scanner under general anesthesia. Patients with MRI-contraindications or undergoing stereotactic procedures other than sole biopsy (e.g. stereotactic catheter placement, aspiration of cyst or abscess, deep brain stimulation) were excluded. As a control sample, we studied the last 100 stereotactic biopsies performed prior to the installation of the intraoperative scanner. Altogether, we analyzed a number of 600 procedures performed on 592 patients from 2007 to 2016.

### Stereotactic imaging

A stereotactic head-frame was mounted in the OR after induction of general anesthesia. Surgeons had the choice between three different frames: a titanium frame (*Stryker-Leibinger GmbH & Co*. *KG*, Freiburg, Germany), a legacy carbon frame (*Stryker-Leibinger*, v.s.) or a ceramic frame (*inomed Medizintechnik GmbH*, Emmendingen, Germany).

**Fig 1:**
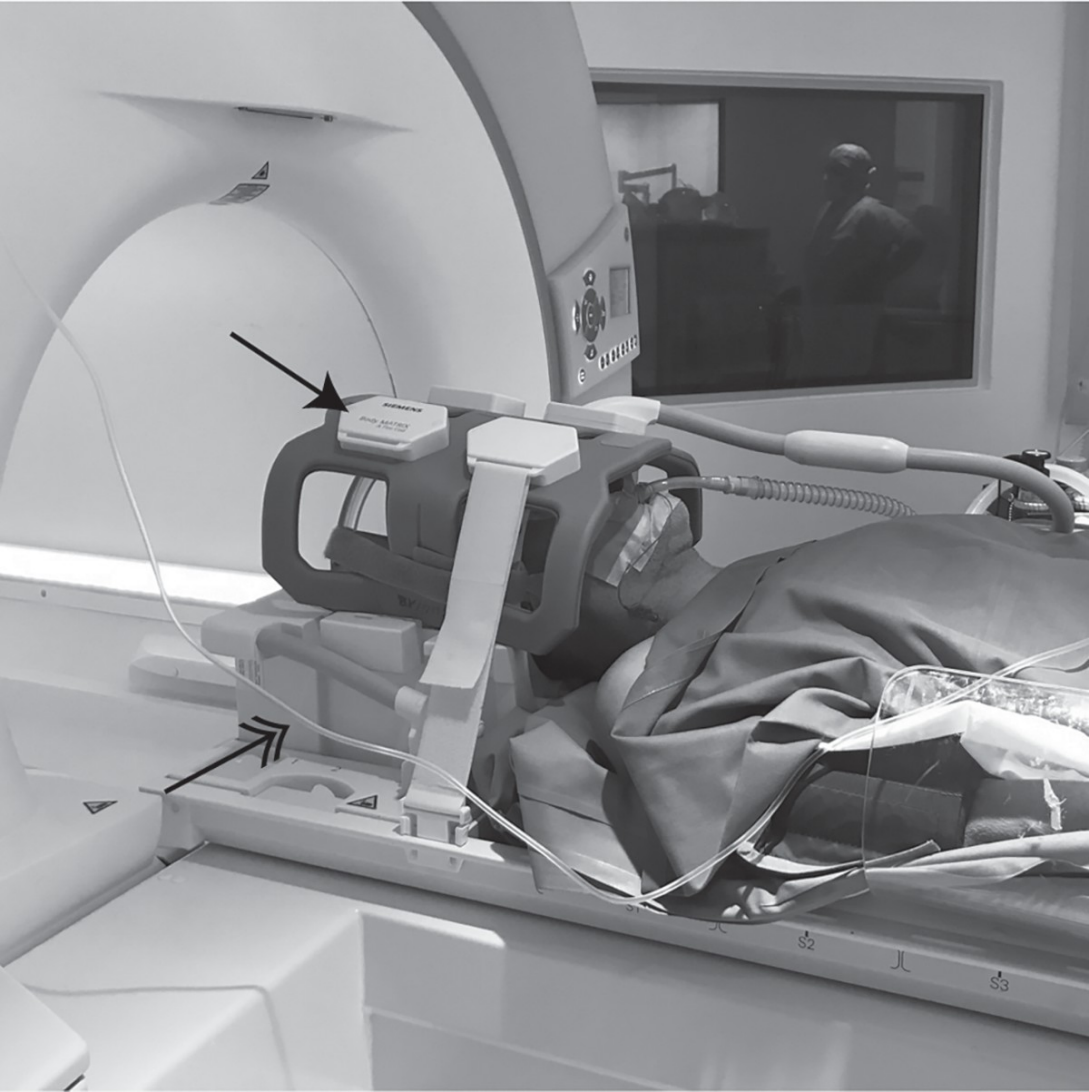
Positioning of the patient in the iMRI-scanner and setup of coils. The head of the patient with mounted frame and localizers is placed into the lower half of the 4-channel send/receive coil (double arrow). Due to the fact that the standard head coil (upper and lower part) does not accommodate the whole package, a flexible coil is used as upper half (black single arrow). This coil assembly was tested extensively prior to commissioning of the iMRI and yields good signal-to-noise ratios.

**Fig 2:**
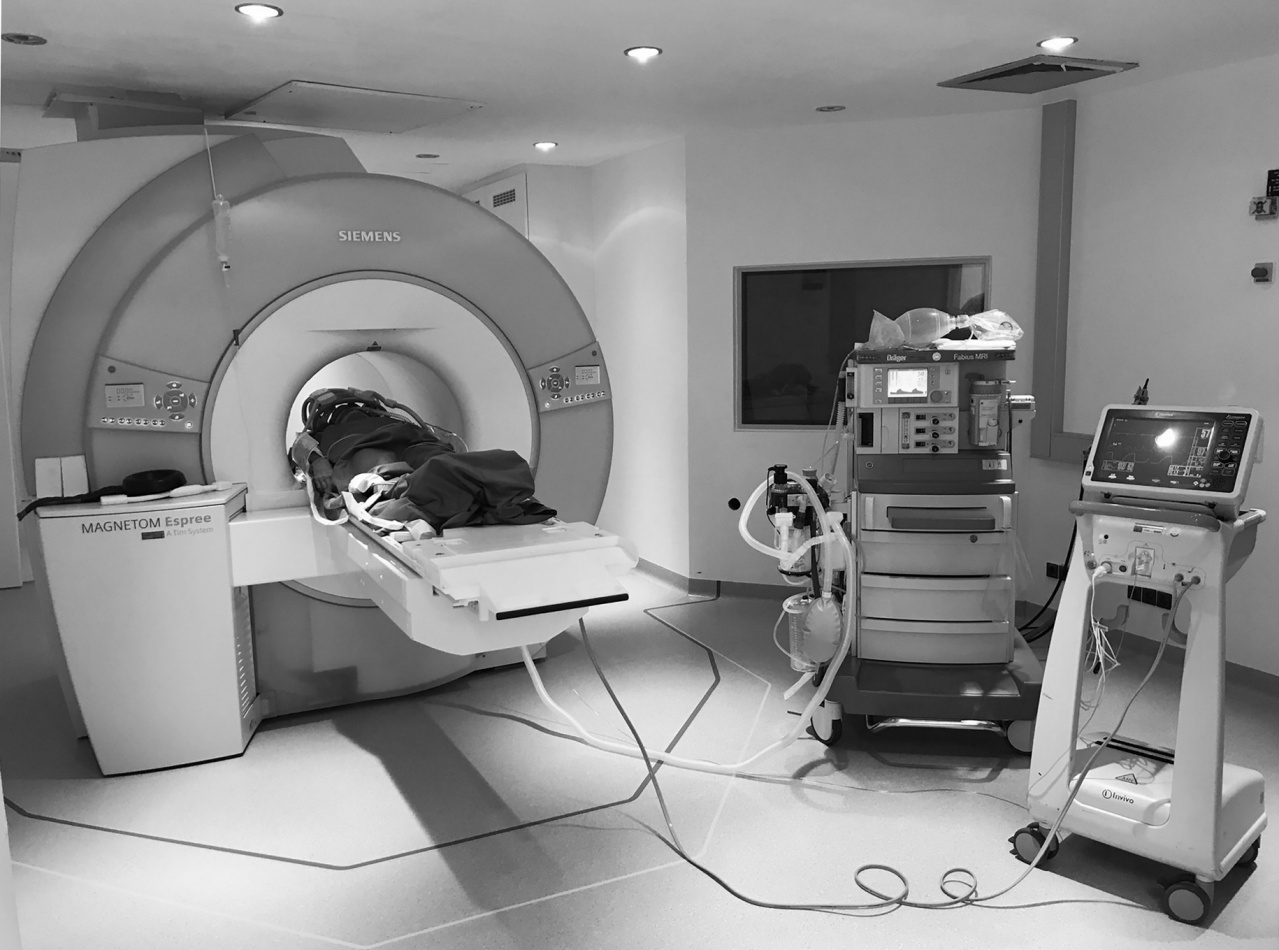
Setup of anesthesia equipment in the iMRI scanner. The MR-capable ventilator (Dräger Fabius MRI, Drägerwerke, Lübeck, Germany) is positioned outside the 30 mT area (dark line) while the monitoring hardware has to be kept outside the 20 mT area (light yellow line).

Patients were then transferred to the iMRI (Magnetom Espree 1.5T, *Siemens Healthcare GmbH*, Erlangen, Germany) in supine position. The head was placed in the bottom half of the standard head send/receive coil. The upper part was enclosed with a flexible 4-channel coil (Body Matrix Coil, Siemens Healthcare GmbH, v.s.) fixated with Velcro straps to accommodate the patient’s head and the attached frames and localizers (Fig 1). The setup of anesthesia equipment in the iMRI scanner is shown in Fig 2.

To obtain a stereotactic image dataset, surgeons had the choice between a T1-MPRAGE or a T1-VIBE isotropic (1 mm voxels) contrast-enhanced (T1-CE) sequence as primary stereotactic imaging sequence. Following T1-CE imaging, additional imaging (e.g. T2, FLAIR, SWI) was performed where deemed necessary for stereotactic planning. After image data acquisition, the distortion-correction algorithm of the scanner was applied.

In the control cohort, stereotactic imaging was performed using contrast-enhanced intraoperative CT (iCT, 1 mm slices) in 75 cases. In cases were iCT was not desired (e.g. due to radiation exposure in children) or was not readily available, the patient was transferred to the department of neuroradiology and stereotactic MRI (5 cases) or CT (20 cases) imaging was performed outside the OR under general anesthesia prior to the stereotactic procedure.

### Stereotactic planning and image fusion

Target selection and trajectory planning was performed with the INOMED Planning System (Version 4 and 5, *inomed Medizintechnik GmbH*, Emmendingen, Germany).

### Surgical technique

The procedures in the iMRI cohort were performed by a total of five neurosurgeons. KK and JON performed more than 80% of the biopsies. All surgeons adhered to the same surgical technique. Surgeons had the choice between a center-of-arc stereotactic device (Zamorano-Duchovny ZD-System) or a high-precision system (Riechert-Mundinger-System, all inomed GmbH, v.s.), which was used primarily for brainstem biopsies via a transfrontal trajectory.

Following trajectory planning, the stereotactic device was assembled and target coordinates were validated using a stereotactic target simulator (*inomed Medizintechnik GmbH*, Emmendingen, Germany). After burr-hole trephination and opening of the dura, specimens were taken along the trajectory down to the target point in one millimeter steps using a 1-mm microforceps. In case of bleeding from the biopsy site, gentle irrigation was applied and the sheath positioned at the bleeding site waiting for the bleeding to cease. The cannula was removed after the last specimen was taken, the burrhole closed with a gelatine sponge and the skin closed in usual fashion.

### Postoperative care and control imaging

After removal of the stereotactic frame, patients were extubated in the operation room and neurologically examined in the OR and/or the recovery room. In case of significant intraoperative bleeding, postoperative neurologic deficit (or worsening of preoperative deficit) or any other condition of concern for the performing surgeon, a CT scan of the brain was performed postoperatively.

### Data collection

Clinical data of all stereotactic procedures were extracted from the clinical information system. The admission, operation and discharge reports as well as pathology results of all eligible patients were successfully retrieved and made available for electronic keyword pattern searching. The Picture Archiving and Control System (PACS) was queried to obtain all relevant intra- and postoperative imaging.

All cases and images were carefully reviewed by the authors (JON, BC, BY) for complications, neurological performance scores and diagnostic yield. The modified Rankin Scale (mRS) and the modified National Institute of Health Stroke Scale (mNIHSS) at admission and discharge were determined independently by two authors (JON, BY) from the clinical documents available. A major neurologic complication was defined as any increase in mRS from admission to discharge, whereas any increase in mNIHSS without concurrent increase in mRS was considered to be a minor neurologic deficit. Ambiguous or presumably non-diagnostic cases were reviewed by two authors (JON, BC) together with a neuropathologist (AVD) and all of these patients or primary caregivers were successfully contacted for clinical follow-up.

### Statistical analysis

Statistical analysis of study data was performed using IBM® SPSS® Statistics 25 (IBM Corp., Armonk, USA). The relevant significance level for statistical testing was set to P = 5%. Fisher’s exact or the chi-square test was used for categorical variables while the Student t-test or one-way Analysis of Variance (ANOVA) were used for continuous data. Ninety-five confidence intervals (CI) are displayed in square brackets. CIs of binomial proportions were estimated by the method of Clopper and Pearson. The meta-analysis of complication rates was performed with R (Version 3.5.0) using the ‘meta’-Package [5] (Version 4.9–1). A random effects model using the inverse variance method with Clopper-Pearson confidence interval for individual studies was used.

### Ethics

The retrospective collection and analysis of patient data were approved by the Institutional Review Board of the University of Heidelberg and patient consent was waived. The individual shown in Figs 1 and 2 in this manuscript has given written informed consent (as outlined in PLOS consent form) to publish these case details.

## Results

### Demographics and preoperative assessment

Both cohorts showed no significant difference in terms of patient age, gender, preoperative neurologic deficit (mRS and mNIHSS) and anesthesia risk assessment score (ASA) [6]. Preoperative clinical characteristics of both groups are summarized in Table 1. The table shows homogeneity of both cohorts (iMRI vs. Control) with respect to demographics, preoperative neurologic status and anesthesia risk assessment.

**Table 1:** Demographics of subjects in both study cohorts.

		iMRI (n = 500)		Control (n = 100)		p
**Collection period**		06/2009–03/2016 (81 months)		04/2008–06/2009 (15 months)		
**Age (Years)**	Range	1–89		2–83		.26^a^
	Mean	55.4		53.1		
	Median	58		55		
**Gender**	Female	231	46%	49	49%	.66^b^
	Male	269	54%	51	51%	
**mRS**	0	255	51%	47	47%	.53^b^
	1	89	18%	16	16%	
	2	85	17%	19	19%	
	3	45	9%	15	15%	
	4	22	4%	3	3%	
	5	4	1%	0	0%	
**mNIHSS**	Range	0–35		0–15		.09^c^
	Median	0		2		
**ASA**	I	33	7%	10	10%	.17^b^
	II	267	53%	47	47%	
	III	188	38%	43	43%	
	IV	12	2%	0	0%	
	V	0	0%	0	0%	

^a^ Independent samples t-test

^b^ Fisher’s exact test

^c^Mann-Whitney U-test

mRS = modified Rankin Scale. mNIHSS = modified National Institute of Health Stroke Scale. ASA = American Society of Anesthesiologists Physical Status Classification System. iMRI = intraoperative MRI

### Stereotactic imaging and planning

In the control cohort, iCT was used in 75% of the cases while non-intraoperative CT and MRI were used in 20% and 5% of the cases, respectively. No safety-related events were reported during the imaging of the intubated and ventilated patients in both cohorts.

Multimodal image fusion with preoperative MRI was used routinely (87%) in the control cohort while it constituted an exception (1%) in the iMRI cohort (Fisher’s exact test, P < .001). Fusion of intraoperative MRI-sequences was swiftly achieved in all cases due to the fact that all sequences shared a common coordinate system and no patient movements did occur during image acquisition under general anesthesia. FET-PET image fusion was only performed in the iMRI cohort (11 cases, 2%).

Table 2 summarizes the brain region targeted during the FBSB procedure. The frontal lobe was the most frequent target area in both groups followed by the temporal lobe. While most biopsies targeted supratentorial lesions, infratentorial targets were more frequent in the iMRI group (10.8% vs. 4.0%, Fisher’s exact test, P = .04).

**Table 2:** Location of biopsy targets.

Target location		iMRI (n = 500)		Control (n = 100)	
		n	%	n	%
**Supra-tentorial**	Frontal lobe	164	32.8%	37	37.0%
	Temporal lobe	78	15.6%	22	22.0%
	Parietal lobe	25	5.0%	9	9.0%
	Occipital lobe	79	15.8%	8	8.0%
	Insula	11	2.2%	3	3.0%
	Basal ganglia	43	8.6%	10	10.0%
	Diencephalon	39	7.8%	6	6.0%
	Mesencephalon	7	1.4%	1	1.0%
	**Total**	**446**	**89.2%**	**96**	**96.0%**
**Infra-tentorial**	Pons	28	5.6%	1	1.0%
	Cerebellum	23	4.6%	2	2.0%
	Medulla oblongata	3	0.6%	1	1.0%
	**Total**	**54**	**10.8%**	**4**	**4.0%**

iMRI = intraoperative MRI

### Surgical procedures

Intraoperative CT was slightly, but significantly faster than iMRI: the mean procedure duration, defined as the time spent between mounting- and unmounting of the stereotactic frame, was 124 [121, 127] minutes using iMRI while procedures based on iCT took 112 [106, 118] minutes on average. Imaging performed outside the OR considerably increased mean procedure time to 160 [149, 171] minutes with CT imaging or even 200 [160, 238] minutes with MRI.

**Fig 3:**
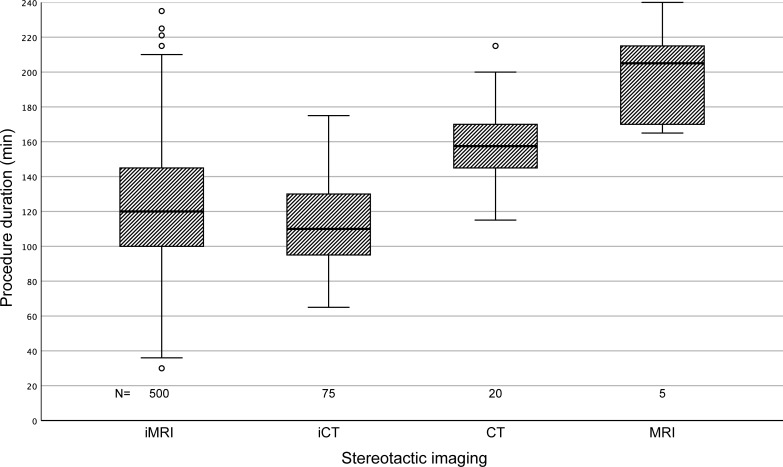
Procedure duration stratified by imaging modality. The duration of the procedures (time spent in the stereotactic frame) was significantly influenced by the imaging modality used to acquire the stereotactic dataset (One-way ANOVA, p < .000). Post-hoc testing (LSD-Bonferroni) showed significant differences between each individual imaging modality with exception of the CT/MRI pair. Procedures performed with intraoperative CT (iCT) required the least amount of time (median 110 min) followed by iMRI-based biopsies in second place (median 120 min). Imaging performed outside the OR (CT and MRI) considerably increased overall procedure time.

The overall differences in procedure duration were statistically significant (one-way ANOVA, P < 0.001) between all four imaging modalities. Post-hoc testing (LSD-Bonferroni, α = 5%) showed significant differences between individual imaging modalities with exception of the CT/MRI pair due to the high variance and low sample size in the latter group (Fig 3).

The number of specimens taken (16 on average) did not differ significantly between both cohorts (unpaired t-test, P = .13, *iMRI*: 3–32, CI [16, 17]; *Control*: 4–31, CI [15, 17]). Univariate linear regression showed no relevant influence of the number of specimens on total procedure time. Furthermore, univariate logistic regression showed no significant correlation between number of specimens taken and morbidity or mortality (P = .48). In the historic cohort, intraoperative examination was used occasionally (25.0% of cases) and did not increase average procedure time (CI [–24, 9], unpaired t-test, P = .35). With the installation of the iMRI, the necessary facilities to perform intraoperative examination were removed from the OR. In iMRI cases where intraoperative examination of specimens was required (4.2% of cases), intraoperative diagnosis had to be made outside the OR and caused a significant increase in procedure time of 29 minutes on average (CI [14, 45], unpaired t-test, P < .001).

### Postoperative imaging and neurologic outcome

CT scanning within seven days post-biopsy was performed more frequently in the iMRI cohort (125 cases, 24.8%) than in the historical controls (15 cases, 15.0%, Fisher’s exact test, P = 0.04). Twenty-two (iMRI) respectively two (control) individuals showed postoperative neurologic symptoms necessitating postoperative imaging. In all remaining cases, imaging was initiated on behalf of the performing surgeons or ward physicians.

Two patients in each group did not receive postoperative CT in spite of neurologic deterioration, because the worsening of pre-existing symptoms was considered to be too minor to warrant imaging. Both patients recovered completely until discharge. Clinically silent hematomas were detected in seven patients (1.4%) in the iMRI-group and two patients (2%) in the historical cohort.

Three fatal cases were encountered in the iMRI cohort due to intratumoral hemorrhage following biopsy. Histological diagnosis revealed glioblastoma in all three cases and life-sustaining therapy was finally withdrawn in the comatose patients within a few days post-surgery. A more detailed account of all cases with postoperative neurologic deterioration is given in the S1 Table.

In summary, new or aggravated neurological symptoms persisting until hospital discharge were encountered in 24 (4.8%) respectively 4 cases (4%). The observed differences between groups were not statistically significant (Fisher’s exact test, P = .69). Tables 3 and 4 summarize neurologic outcomes as well as frequency and result of postoperative imaging.

**Table 3:** Postoperative imaging, hematoma and neurologic status in the iMRI cohort.

iMRI				Postoperative deterioration				Total
				Death	Major	Minor	None	
**Postoperative CT**	No			0	0	2	374	**376**
	Yes	**Hematoma**	No	0	8	7	95	**110**
			Yes	3	4	0	7	**14**
**Total**				**3**	**12**	**9**	**476**	**500**

iMRI = intraoperative MRI

**Table 4:** Postoperative imaging, hematoma and neurologic status in the control cohort.

Control				Postoperative deterioration				Total
				Death	Major	Minor	None	
**Postoperative CT**	No			0	0	2	83	**85**
	Yes	**Hematoma**	No	0	1	1	11	**13**
			Yes	0	0	0	2	**2**
**Total**				**0**	**1**	**3**	**96**	**100**

iMRI = intraoperative MRI

### Histological results

The two groups were homogenous with respect to lesion etiologies (Fisher’s exact test, P = .79), but there was a statistically significant difference in the frequencies of individual histological diagnoses (Chi-square test, P = 0.04, S2 Table).

The three most common tumor entities encountered were glioblastoma (43.4% iMRI vs. 36.0% control) followed by diffuse astrocytoma (10.2% vs. 19.0%) and lymphoma (11.2% vs. 14.0%). The diagnosis of brain metastasis was uncommon in both groups (1.2% vs. 2.0%).

Diagnosis was incorrect or missed for 16 cases (3.2%) in the iMRI group and in 4 cases (4.0%) in the control group. This difference was not statistically significant (Fisher’s exact test, P > 0.99).

### Summary of complications

Table 5 summarizes the overall complication rates and gives confidence intervals for each undesired event. Mortality was 0.6% [0.12%, 1.74%] in the iMRI and 0.0% [0.0%, 3.6%] in the control group. Procedure-related morbidity (neurologic deficit & infection) was 5.4% [3.6%, 7.8%] in the iMRI and 6.0% [2.2%, 12.6%] in the control group. No significant differences in event rates were noted between both groups.

**Table 5:** Summary of complications.

	iMRI (n = 500)				Control (n = 100)				
	n	%	95% C.I.*		n	%	95% C.I.*		p**
			lower	upper			lower	upper	
**Mortality**	**3**	**0.6%**	0.12%	1.74%	**0**	**0.0%**	0.00%	3.62%	.99
**Major deficit**	**12**	**2.4%**	1.25%	4.15%	**1**	**1.0%**	0.03%	5.45%	.70
**Minor deficit**	**9**	**1.8%**	0.83%	3.39%	**3**	**3.0%**	0.62%	8.52%	.43
**Wound infection**	**6**	**1.2%**	0.44%	2.59%	**2**	**2.0%**	0.00%	3.62%	.62
**Deep infection / abscess**	**0**	**0.0%**	0.00%	0.74%	**0**	**0.0%**	0.00%	7.04%	.99
**Missed / incorrect diagnosis**	**16**	**3.2%**	1.84%	5.14%	**4**	**4.0%**	1.10%	9.93%	.99

iMRI = intraoperative MRI

*Clopper-Pearson

**two-sided Fisher’s exact test

## Discussion

In this study, we compared a series of five hundred iMRI-based FBSBs with a historic control series of one hundred biopsies, mostly conducted using iCT. As hypothesized, we found no significant differences in mortality, morbidity and diagnostic yield between both groups.

In order to compare the rates of undesired events in our sample with the literature, we conducted a meta-analysis of 24 prior FBSB series [7–30]. The scope of the literature research was limited to studies published in this millennium to mitigate possible effects of recent technical advancements in stereotactic hardware, imaging and planning. The large series of Lunsford et al. [31], although published in 2008, was not included due to the large retrospective collection period of 28 years.

For the meta-analysis, a random-effects model with an inverse variance method was used (forest plots Figure A-C in S1 Fig). The mortality of 0.6% [0.12%, 1.74%] found in our iMRI-group is in-line with the average mortality of 0.9% [0.6%, 1.4%] determined by our meta-analysis, which included more than ten thousand cases from the literature. The risk of neurologic deficit persisting until hospital discharge of 4.2% [2.6%, 6.4%] in our series is equivalent to the proportion in the meta-analysis of 4.1% [3.0%, 5.6%], even though our definition of “neurologic deficit” based on mRS and NIHSS might be stricter than the definition used in some other studies.

Although intraoperative examination of specimens was used rarely in combination with iMRI-based FBSB, the rate of missed or incorrect diagnosis of 3.2% [1.8%, 5.1%] was lower than in the control group (4.0% [1.1%, 9.9%]) or the meta-analysis (5.3% [3.9%, 7.1%]).

While Tilgner et al. have established the high validity of intraoperative diagnosis by showing more than 90% correlation with final diagnosis [16], it remains unclear whether this leads to lower complication rates or higher diagnostic yields because surgeons might take fewer or more biopsy bits depending on intraoperative examination. While Jain et al. [32] did report a positive association between bits taken and diagnostic accuracy and Sawin et al. [33] as well as McGirt et al. [34] observed higher morbidity in subgroups of patients with a higher mean number of biopsy attempts, while others–including us—have found no relationship between the number of biopsy attempts and mortality or morbidity [8,9,13,35]. It seems rather unlikely that the marginal risk of every other biopsy bit will ever be established even in much larger series owing to the low overall incidence of adverse events in these types of procedures. Nonetheless, Livermore [36] et al. have argued strongly in favor of intra-operative histological analysis, especially in cases of broad pre-biopsy differential diagnosis. In our opinion, the overall effectiveness of *routine* intraoperative examinations of stereotactic biopsies remains to be questioned and we have limited its use to selected cases (4% in our iMRI cohort) while maintaining a favorable diagnostic yield and a low morbidity and mortality.

Only limited data on the duration of FBSB procedures have been published so far. A series of 79 frame-based stereotactic biopsies by Dorward et al. [11] represents the best benchmark available as of today. In this series, both imaging and surgical procedure were performed under general anesthesia for a mean time of 127 [120, 134] minutes. The average duration of 124 [121, 127] minutes achieved in our iMRI cohort compares favorably to this benchmark of a predominantly (90%) CT-based series. In a series more balanced with respect to imaging modality (n = 213, 44% CT vs. 56% MRI), but performed solely under local anesthesia, a total procedure time of 114 [109, 119] minutes was reported [15]. In our view, this relatively small gain in procedure time does not warrant preferring local to general anesthesia when intraoperative imaging (iCT or iMRI) is available.

Frame-less stereotactic biopsies (FLSBs), i.e. diagnostic procedures with the application of an optical navigation system, have been directly compared to FBSBs by several authors [11,15,23,29,36–38]. They have generally been reported to be as effective and safe as FBSBs while consuming less time in the OR with the exception of the series published by Smith et al. [15], which found a significantly longer total procedure time for FLSB compared to FBSB (185 vs. 114 minutes). We performed a meta-analysis of all four [11,15,23,38] of the above-mentioned studies that have reported mean and standard deviations for the total procedure time. The random effects model yielded a 14.2 [-36.3, 64.6] minutes time gain when comparing FLSB to FBSB (S2 Fig).

As the precision of FLSB does not only depend on rigorous function of the optical system but also on the most accurate registration of the head position in space, there is still reluctance to use FLSB for small and deep-seated lesions. In our opinion, there is still insufficient data to conclude that navigation-guided procedures are safe in these kinds of lesions. In the recent study of Georgiopoulos et al. [38], the smallest maximum diameter of targets was 15 mm, a size that can be regarded as rather large compared to the (sub-)millimeter tolerances reliably achieved by frame-based procedures in the placement of deep-brain stimulation electrodes.

In summary, we conclude that our iMRI-based FBSB approach offers a well-balanced compromise of operation time, safety, effectiveness, precision and–last but not least—patient comfort due to the use of generel anesthesia during the whole operative procedure.

Our study has several limitations. It is retrospective in nature and therefore prone to selection bias and may contain clinical data of reduced quality. In our effort to generate a large sample size, a noticeable imbalance of group sizes had to be accepted: the control group is only one-fifth the size of the study group. A larger control group would have necessitated going back further in time and including cases with different surgeons, surgical techniques and devices (i.e. frames and planning software).

Due to the retrospective analysis, clinical data on neurological performance had to be taken from clinical records. The mRS and mNIHSS scores were independently determined from the records by two authors and a consensus was reached on any disagreement. It has been shown that the mNIHSS can be reliably estimated from medical records [39]. Our definition of major and minor neurological deficit along the mRS and mNIHSS scores increases interobserver reliability.

Routine postoperative CT scanning was not performed in patients. As a consequence, we might have missed asymptomatic hemorrhages. In a series of 355 consecutive biopsies with mandatory postoperative scanning, Grossman et al. [13] reported an incidence of 3.4% asymptomatic hemorrhages. In our series, we found asymptomatic hemorrhages in 7.8% of the asymptomatic patients scanned postoperatively (115 total, c. Table 3). Nevertheless, we do not think that this higher incidence of asymptomatic hemorrhages warrants routine scanning as none of the patients deteriorated neurologically in the following time.

## Conclusion

Following our analysis, we conclude that FBSB using iMRI under general anesthesia is a safe and effective procedure and is equivalent to traditional stereotactic workflows with respect to complication rate and diagnostic yield. It reduces radiation exposure while offering increased patient and surgeon comfort by offering the possibility to perform the whole procedure in a one-stop-fashion under general anesthesia. It seems evident that procurement of iMRI solely for FBSB is not cost-effective, but the integration of iMRI into modern neurosurgical theatres is increasing and we recommend its routine use for stereotactic procedures where already available.

## Supporting information

S1 Table Clinical features of patients with neurologic deterioration associated with biopsy procedure. (DOCX)

S2 Table Histologic results. The three most common tumor entities encountered in both groups were glioblastoma followed by diffuse astrocytoma and lymphoma. The differences in etiologies (glial tumors, non-glial tumors, infection etc…) was not statistically significant (Fisher’s exact test, P = .79).
(DOCX)

S1 Fig Meta-analysis of mortality, morbidity and diagnostic yield in stereotactic biopsy series. Forest plots of random effects model meta-analysis (inverse variance method with Clopper-Pearson confidence interval for individual studies) of death (Figure A in S1 Fig), new or worsened neurologic deficit (Figure B in S1 Fig) and missing diagnosis (Figure C in S1 Fig) following FBSB. To mitigate the effect of technical advancements in imaging and planning, only studies published in this millennium were included.
(EPS)

S2 Fig Meta-analysis of total procedure time in studies directly comparing frame-based (FBSB) to frame-less (FBSL) biopsy procedures. (EPS)

S1 File STROBE checklist. (DOCX)

S2 File Relevant underlying source data. (CSV)
