# Supplementary material for: Frame-based stereotactic biopsies using an intraoperative MR-scanner are as safe and effective as conventional stereotactic procedures
Source: PLoS One. 2018 Oct 23;13(10):e0205772. doi: 10.1371/journal.pone.0205772 (PMC6198960; doi:10.1371/journal.pone.0205772)
Supplement: S1 Table — (DOCX) [file pone.0205772.s001.docx]

| **Cohort** | **Sex** | **Age** | **Histo** | **Location** | **Outcome** | **Cause** | **Clot volume (ml)** | **Neurologic deterioration** | **mRS** | | **mNIHSS** | |
| --- | --- | --- | --- | --- | --- | --- | --- | --- | --- | --- | --- | --- |
|  |  |  |  |  |  |  |  |  | **pre** | **post** | **pre** | **post** |
| Historic | ♀ | 54 | IS | Frontal lobe | Major deficit | Ischemic stroke | - | Aphasia & reduced alertness | 1 | 3 | 0 | 7 |
| Historic | ♂ | 58 | GBM | Parietal lobe | Minor deficit | Edema | - | Mild hemiparesis | 1 | 1 | 2 | 7 |
| Historic | ♀ | 70 | ENC | Basal ganglia | Minor deficit | Edema | - | Worsening of hemiparesis | 3 | 3 | 10 | 13 |
| Historic | ♀ | 52 | DA | Brainstem | Minor deficit | Edema | - | Hypesthesia left hand & mild CN VI palsy | 2 | 2 | 5 | 8 |
| iMRI | ♂ | 66 | GBM | Brainstem | Death | Hemorrhage | 10 | Comatose -> withdrawal of therapy | 0 | 6 | 0 | 5 |
| iMRI | ♀ | 2 | GBM | Brainstem | Death | Hemorrhage | 11 | Comatose -> withdrawal of therapy | 0 | 6 | 0 | 0 |
| iMRI | ♂ | 72 | GBM | Basal ganglia | Death | Hemorrhage | 43 | Comatose -> withdrawal of therapy | 3 | 6 | 5 | 35 |
| iMRI | ♀ | 62 | LYM | Basal ganglia | Major deficit | Hemorrhage | 1 | Hemiplegia | 0 | 4 | 0 | 8 |
| iMRI | ♂ | 76 | IS | Frontal lobe | Major deficit | Ischemic stroke | - | Hemiparesis, dysphagia & neglect | 0 | 4 | 0 | 7 |
| iMRI | ♀ | 75 | GBM | Temporal lobe | Major deficit | Hemorrhage | 5 | Hemiparesis & neglect | 0 | 3 | 0 | 6 |
| iMRI | ♀ | 82 | DA | Frontal lobe | Major deficit | Ischemic stroke | - | Upper extremity paresis | 2 | 4 | 4 | 9 |
| iMRI | ♀ | 54 | LYM | Frontal lobe | Major deficit | Disease progression | - | Reduced alertness, hemiparesis & aphasia | 3 | 4 | 6 | 12 |
| iMRI | ♀ | 74 | GBM | Basal ganglia | Major deficit | Edema | - | Worsening of hemiparesis | 2 | 3 | 3 | 6 |
| iMRI | ♂ | 56 | GBM | Temporal lobe | Major deficit | Hemorrhage | 30 | Coma & hemiparesis -> good recovery later | 0 | 2 | 0 | 5 |
| iMRI | ♂ | 73 | PA | Brainstem | Major deficit | Hemorrhage | 1 | Worsening of hemiparesis | 3 | 4 | 9 | 12 |
| iMRI | ♀ | 56 | GBM | Basal ganglia | Major deficit | Edema | - | Worsening of hemiparesis | 2 | 3 | 4 | 6 |
| iMRI | ♀ | 78 | GBM | Parietal lobe | Major deficit | Edema | - | Upper extremity paresis | 3 | 4 | 7 | 9 |
| iMRI | ♀ | 40 | DA | Brainstem | Major deficit | Edema | - | Mild hemiparesis & diplopia | 0 | 1 | 0 | 2 |
| iMRI | ♀ | 77 | LYM | Basal ganglia | Major deficit | Edema / disease progression | - | Upper extremity paresis | 2 | 3 | 5 | 7 |
| iMRI | ♂ | 25 | AA | Brainstem | Minor deficit | Edema | - | Facial palsy & diplopia | 1 | 1 | 2 | 7 |
| iMRI | ♂ | 71 | AA | Brainstem | Minor deficit | Edema | - | Dysphagia | 2 | 2 | 2 | 5 |
| iMRI | ♂ | 32 | DA | Frontal lobe | Minor deficit | Edema | - | Mild vision disturbance | 1 | 1 | 2 | 4 |
| iMRI | ♀ | 67 | NONE | Basal ganglia | Minor deficit | Edema / unknown | - | Worsening of hemiparesis | 2 | 2 | 5 | 7 |
| iMRI | ♀ | 78 | GBM | Temporal lobe | Minor deficit | Edema | - | Worsening of dysphasia | 2 | 2 | 4 | 6 |
| iMRI | ♀ | 75 | INF | Frontal lobe | Minor deficit | Edema / unknown | - | Decline of fine motor skills | 3 | 3 | 5 | 7 |
| iMRI | ♂ | 63 | VAS | Brainstem | Minor deficit | Disease progression | - | Worsening of hemiparesis | 2 | 2 | 4 | 5 |
| iMRI | ♀ | 68 | DA | Frontal lobe | Minor deficit | Diffuse hemorrhage | - | Worsening of hemiparesis | 2 | 2 | 3 | 4 |
| iMRI | ♀ | 61 | AA | Brainstem | Minor deficit | Edema | - | Worsening of facial palsy | 3 | 3 | 7 | 8 |

IS=Ischemic stroke, GBM=Glioblastoma, ENC=Encephalitis, DA=Diffuse astrocytoma, LYM=Lymphoma, PA=Pilocytic astrocytoma, AA=Anaplastic astrocytoma,

INF =Infection/abscess

**S1 Table**

**Clinical features of patients with neurologic deterioration associated with biopsy procedure**
