# Supplementary material for: Frame-based stereotactic biopsies using an intraoperative MR-scanner are as safe and effective as conventional stereotactic procedures
Source: PLoS One. 2018 Oct 23;13(10):e0205772. doi: 10.1371/journal.pone.0205772 (PMC6198960; doi:10.1371/journal.pone.0205772)
Supplement: S2 Table — The three most common tumor entities encountered in both groups were glioblastoma followed by diffuse astrocytoma and lymphoma. The differences in etiologies (glial tumors, non-glial tumors, infection etc…) was not statistically significant (Fisher’s exact test, P = .79). (DOCX) [file pone.0205772.s002.docx]

|  | | iMRI  (n=500) | | Control  (n=100) | |
| --- | --- | --- | --- | --- | --- |
|  |  | n | % | n | % |
| Glial tumors | Glioblastoma | 217 | 43.4% | 36 | 36.0% |
|  | Diffuse astrocytoma | 51 | 10.2% | 19 | 19.0% |
|  | Anaplastic astrocytoma | 49 | 9.8% | 6 | 6.0% |
|  | Pilocytic astrocytoma | 14 | 2.8% | 2 | 2.0% |
|  | Oligodendroglioma | 6 | 1.2% | 0 | 0.0% |
|  | Medulloblastoma | 6 | 1.2% | 0 | 0.0% |
|  | Ganglioglioma | 2 | 0.4% | 1 | 1.0% |
|  | Anaplastic oligodendroglioma | 3 | 0.6% | 0 | 0.0% |
|  | Diffuse midline-glioma | 2 | 0.4% | 0 | 0.0% |
|  | Anaplastic oligoastrocytoma | 1 | 0.2% | 1 | 1.0% |
|  | Anaplastic ependymoma | 2 | 0.4% | 0 | 0.0% |
|  | PNET | 0 | 0.0% | 1 | 1.0% |
|  | Pleomorphic xanthoastrocytoma | 1 | 0.2% | 0 | 0.0% |
|  | Ependymoma | 0 | 0.0% | 1 | 1.0% |
|  | Anaplastic pleomorpic xanthoastrocytoma | 1 | 0.2% | 0 | 0.0% |
| Non-glial tumors | Lymphoma | 56 | 11.2% | 14 | 14.0% |
|  | Metastasis | 6 | 1.2% | 2 | 2.0% |
|  | Germinoma | 3 | 0.6% | 0 | 0.0% |
| Infection | Progressive multifocal leukencephalopathia | 3 | 0.6% | 1 | 1.0% |
|  | Fungal infection | 2 | 0.4% | 0 | 0.0% |
|  | Viral encephalitis | 0 | 0.0% | 1 | 1.0% |
|  | Toxoplasmosis | 1 | 0.2% | 0 | 0.0% |
|  | Bacterial abscess | 1 | 0.2% | 0 | 0.0% |
| Inflammation | Encephalitis disseminata | 18 | 3.6% | 4 | 4.0% |
|  | Vasculitis | 9 | 1.8% | 0 | 0.0% |
|  | ADEM | 3 | 0.6% | 4 | 4.0% |
|  | Sarcoidosis | 1 | 0.2% | 0 | 0.0% |
| Neurodegenerative diseases | Leukodystrophia | 1 | 0.2% | 0 | 0.0% |
| Other | CNS-tissue | 34 | 6.8% | 4 | 4.0% |
|  | Infarction | 4 | 0.8% | 2 | 2.0% |
|  | Radionecrosis | 1 | 0.2% | 1 | 1.0% |
|  | Toxic leukencephalopathia | 1 | 0.2% | 0 | 0.0% |
|  | Epidermoid | 1 | 0.2% | 0 | 0.0% |

iMRI = intraoperative MRI

**Table S2. Histologic Results.**

The three most common tumor entities encountered in both groups were glioblastoma followed by diffuse astrocytoma and lymphoma. The differences in etiologies (glial tumors, non-glial tumors, infection etc…) was not statistically significant (Fisher’s exact test, P = .79).
